# Reduction of the Vertebral Bone Mineral Density in Patients with Hodgkin Lymphoma Correlates with Their Age and the Treatment Regimen They Received

**DOI:** 10.3390/cancers14030495

**Published:** 2022-01-19

**Authors:** Nadav Ofshenko, Eyal Bercovich, Tania Mashiach, Michal Weiler-Sagie, Daniela Militianu, Eldad J. Dann

**Affiliations:** 1 Ruth and Bruce Rappaport Faculty of Medicine, Technion, Haifa 3109601, Israel; ofshenko@campus.technion.ac.il; 2 Department of Radiology, Rambam Health Care Campus, Haifa 3109601, Israel; e_bercovich@rmc.gov.il (E.B.); d_militianu@rambam.health.gov.il (D.M.); 3 Quality Assurance Unit, Rambam Health Care Campus, Haifa 3109601, Israel; tania.mashiah@gmail.com; 4 Department of Nuclear Medicine, Rambam Health Care Campus, Haifa 3109601, Israel; m_weiler@rmc.gov.il; 5 Department of Hematology and Bone Marrow Transplantation, Rambam Health Care Campus, Haifa 3109601, Israel; 6 Blood Bank and Apheresis Unit, Rambam Health Care Campus, Haifa 3109601, Israel

**Keywords:** Hodgkin lymphoma, bone mineral density, osteopenia, chemotherapy regimens, steroids, gonadotoxicity, PET/CT

## Abstract

**Simple Summary:**

Hodgkin lymphoma (HL) is considered a largely curable disease (~80%). The young patient age at diagnosis and their long life expectancy make quality-of-life issues, including osteopenia, exceedingly important. This study aimed to assess treatment-related bone mineral density (BMD) changes that are overlooked in this young population. BMD was measured using PET/CT scans. Among 213 patients (median age 29 years), post-treatment BMD reduction of >15% was significantly more common in those aged ≥30 years and was also associated with a cumulative dose of steroids used. At 6 months post-therapy, BMD recovery was observed in ABVD (adriamycin/bleomycin/vinblastine/dacarbazine) treated patients, while individuals receiving EB (bleomycin/etoposide/adriamycin/cyclophosphamide/oncovin/procarbazine/prednisone) regimens demonstrated persistent BMD loss and higher rates of osteopenia. Our findings suggest that steroid use should be minimized and highly gonadotoxic drugs like procarbazine should be substituted with less toxic ones, due to their deleterious effect on BMD. Adequate vitamin D levels should be maintained.

**Abstract:**

Nowadays, Hodgkin lymphoma (HL) has become highly curable. The young age at diagnosis and long life expectancy emphasize the importance of preventing long-term treatment side effects, including bone mineral density (BMD) loss, in these patients. We aimed to evaluate the effects of first-line therapeutic modalities on BMD dynamics in HL patients, intending to identify individuals at risk for osteopenia. Demographics, HL risk factors, treatment, including cumulative steroid doses, and BMD of 213 newly-diagnosed HL patients (median age 29 years), treated at Rambam between 2008–2016, were analyzed. The main chemotherapy regimens applied were: ABVD (adriamycin, bleomycin, vinblastine, dacarbazine) and escalated BEACOPP (EB; bleomycin, etoposide, adriamycin, cyclophosphamide, oncovin, procarbazine, prednisone). BMD was measured using PET/CT scans. BMD loss >15% was revealed in 48% of patients at therapy completion, with osteopenia prevalence of 4% and 14% at baseline and post-therapy, respectively. Cumulative hydrocortisone equivalent doses >3400 mg/m^2^ correlated with significant BMD reduction. Multivariate analysis at 6 months post-therapy identified age ≥30 years and EB-regimens as significant risk factors for BMD decrease >15%. Therapy-related BMD loss is common in HL patients. Its persistence is associated with age ≥30 years and EB treatment. Reduction of cumulative steroid doses and switch to non-gonadotoxic drugs should be considered.

## 1. Introduction

Over the past decades, Hodgkin lymphoma (HL) has become a disease with a high potential of cure [1,2,3,4]. Depending on the HL stage, current treatment protocols provide a 5-year progression-free survival (PFS) of 66–92% and an overall survival (OS) of 84–97% [5,6,7]. Chemotherapy and radiation therapy have significantly improved with time, and have become more efficient and less toxic [8,9]. Despite this progress, HL treatment complications such as pulmonary fibrosis, cardiac disease, secondary malignancies, and infertility remain of significant concern [10,11,12].

Presently, ABVD (adriamycin, bleomycin, vinblastine, dacarbazine) and escalated BEACOPP (bleomycin, etoposide, adriamycin, cyclophosphamide, vincristine, procarbazine, prednisone) are the two backbone chemotherapy protocols employed in HL management. To ameliorate regimen-related toxicity, particularly that of bleomycin, glucocorticoids are used as an integral part of both modalities, according to the Guidelines of the American Society for Clinical Oncology (ASCO) [13]. At the Rambam Health Care Campus, a tertiary care center in the North of Israel, escalated BEACOPP (EB) is generally employed in high-risk HL patients (International Prognostic Score (IPS) ≥3, positive interim PET (PET-2), or a bulky mediastinal mass). However, the duration of the steroid course is reduced to 7 days only instead of the 14 days prescribed in the original protocol by the German Hodgkin Study Group (GHSG) [14,15]. This modification, made in 2002, has aimed to minimize the risk of aseptic necrosis of the head of femur. Glucocorticoids are known to interfere with bone formation and remodeling, which results in an increased fracture risk even for patients receiving prednisolone doses of less than 10 mg/day for extended periods of time. It can also lead to osteopenia and even osteoporosis [16,17,18]. Dual-energy X-ray absorptiometry (DXA) is the gold standard employed for bone mineral density (BMD) measurement. At the same time, since PET/CT imaging is an integral part of staging and follow-up evaluation of HL patients, these scans are being increasingly used as an opportunistic screening approach for BMD assessment [19,20,21,22,23]. This is based on the seminal study by Pickhardt et al. [19] that has compared more than 2000 pairs of CT-DXA exams performed in the same patients (mean age 59 years). In that study, CT attenuation has been measured in Hounsfield units (HU) and the value of 160 HU (T-score ≥ −1.0 SD) has been used to define normal BMD and the threshold of 110 HU (T-score between −1.0 SD and −2.5 SD), has been set to distinguish between normal and osteopenic conditions.

The current study has been designed to assess the impact of different first-line treatment modalities, age, and gender on the vertebral BMD of patients with HL, in order to consider early intervention aiming to preserve the bone tissue in HL survivors.

## 2. Patients and Methods

This retrospective study incorporated newly diagnosed HL patients treated at Rambam between 2007–2016. Baseline patient clinical characteristics and data on the treatment applied, including glucocorticoids, were retrieved from institutional electronic medical records. The study inclusion criteria were: HL histology, availability of baseline and end-of-treatment CT or PET/CT results, and first-line treatment with ABVD or EB. The exclusion criteria were: imaging compatible with HL involvement at the third lumber vertebra (L3) or intravenous use of CT contrast.

### 2.1. Treatment Protocols

Patients were treated according to the National H2 protocol [14]. In brief, individuals with early favorable-risk HL were treated with two ABVD cycles. Patients with a complete metabolic response after two cycles (confirmed by interim PET/CT) underwent involved-site radiation therapy (ISRT); those with positive interim PET/CT results received two additional ABVD cycles followed by ISRT. Patients with early unfavorable-risk HL and negative interim PET/CT were given a total of 4 ABVD cycles + ISRT or 6 ABVD cycles; individuals with positive interim PET/CT were treated with 6 ABVD cycles + ISRT. Patients, defined as having advanced-stage HL with IPS 0–2, initially received two ABVD cycles, while advanced-stage patients with IPS ≥ 3 were initially treated with two EB cycles. Patients with negative interim PET/CT were given four additional ABVD cycles, and those with a positive result had four additional cycles of EB.

According to the National Comprehensive Cancer Network Guidelines, corticosteroids were added to the ABVD regimen as an antiemetic therapy [24], with a dexamethasone dose of 20 mg used until 2012, which was then reduced to 12 mg and given on days 1 and 14 of each cycle.

In our study, prednisone, an integral part of the EB regimen (given for 14 days in the original protocol), was administered only on days 1–7 at a daily dose of 40 mg/m^2^ and dexamethasone was given on day 8 as an antiemetic therapy. Patients were grouped based on the treatment protocol and the number of cycles they received: ABVD × 2–4, ABVD × 6, EB × 2 + ABVD × 4, EB × 4–6 ± ABVD × 2. The cumulative dose of hydrocortisone equivalents (HE), as well as HE given per meter squared (HE/m^2^) of the body surface area, were calculated for each patient. A sub-analysis of a potential correlation between various doses of HE/m^2^ used and vertebral BMD changes was performed.

### 2.2. Computed Tomography

HL staging PET/CT (non-IV contrast CT) scans were used to measure the baseline vertebral BMD at the L3 vertebra level. Each finding was confirmed by an expert radiologist.

The effect of the chemotherapy regimens and glucocorticoids on vertebral BMD was assessed using the end-of-therapy PET/CT scans, and a further comparison was carried out using the scans done 6 months after completion of therapy. Full body PET/CT imaging was performed using the Discovery 690 or Discovery LS system (GE Healthcare, Milwaukee, WI, USA), approximately 60 min after the injection of 0.14 mCi/kg F^18^ FDG.

### 2.3. Hounsfield Unit Measurements

The Hounsfield unit (HU) scale was used to quantify BMD. HU values were correlated with the BMD assessed by DXA [19,22,23,25,26]. High HU values correlated with higher bone density and low HU values correlated with lower bone density. HU measurements were performed on an axial image of the L3 vertebra [19]. For each vertebra, a region of interest (ROI) measurement was taken at three levels: (1) inferior to the superior endplate of L3, (2) at the center of L3, (3) superior to the inferior endplate of L3. To prevent measurement inaccuracy, ROI did not include the cortical bone or blood vessels. The BMD was determined as an average HU value of three ROI measurements. The threshold distinguishing between normal and abnormal BMD was set at 160 HU, as suggested by Pickhardt et al. [19]. The cutoff value of 160 HU does not necessarily apply to 30-year old patients. However, this value was applicable to 81% of our patients for whom the mean value of normal was 167.9 ± 47.2 HU. In the current study, BMD was defined as borderline low if HU values were within one SD lower than normal levels (i.e., 121–159), which added another 15% of patients to those who were not classified as having osteopenia (a total of 96% of patients). Osteopenia was defined as BMD of 80–120 HU, equivalent to a decrease of 1–2.5 SD in the statistical *t*-test. Osteoporosis was defined as BMD of <80 HU, equivalent to a decrease of <2.5 SD in the statistical *t*-test [27].

To assess the incidence of significant BMD loss in HL patients in correlation with their age, gender, and treatment protocols, additional analyses of BMD changes between baseline versus end-of-therapy and baseline versus 6-month post-therapy levels, were performed using a reduction above 15% and above 25% as significant cutoffs.

### 2.4. Statistical Analysis

Differences between treatment groups in terms of demographic variables and corticosteroid dosage were assessed using the Chi-square test. Differences in vertebral BMD pre- and post-therapy were measured using the Mann–Whitney test. Bivariate logistic regression analysis was applied to evaluate the vertebral BMD change between the baseline and end-of-therapy measurements as well as between the baseline and 6-month post-treatment measurements. A BMD decrease of >15% was considered significant. Multivariate logistic regression analysis was used to identify independent factors for the decrease in vertebral BMD. *p*-values ≤ 0.05 were considered statistically significant. SPSS software version 25 was used for the analyses.

## 3. Results

### 3.1. Patient Characteristics and HL Therapy

A total of 248 HL patients with available baseline and end-of-treatment CT scans were identified in the database. Patients who underwent IV contrast CT imaging (*n* = 11) and those receiving corticosteroid treatment for other conditions (*n* = 24) were excluded. Two hundred and thirteen HL patients were included in the final analysis. Their demographic (age, gender) and baseline disease characteristics (stage, risk group), treatment given according to the risk group, including steroids, as well as data on baseline BMD are presented in Table 1. The median age was 29 (range 18–59) years and the male:female ratio was 0.97.

The mean cumulative HE doses per body surface administered per patient are specified in Table 2. The steroid dose differences between ABVD × 2–4 versus ABVD × 6, ABVD × 6 versus EB × 2 + ABVD × 4 and EB × 2 + ABVD × 4 versus EB × 4–6 protocols were highly significant (*p* = 0.000) (Table 2).

### 3.2. Vertebral BMD Dynamics during HL Therapy and Follow-Up

For the 213 evaluable patients, the median vertebral BMD value at baseline was 197 Hounsfield units (HU; 30.5-320) and it decreased to 165 HU (28–277) at the end of therapy (Table 2). The mean (±SD) BMD difference between measurements at these two time points was 16.4 ± 11.2% [median 15 (0.0–54.6)]. At baseline, 8 patients (4%) had osteopenia and one patient had osteoporosis. Measurements made at the end of therapy and 6 months post-treatment revealed osteopenia in 12% of patients and osteoporosis in 2% of patients at both time points (Table 1 and Table 3). A highly significant difference in the BMD values between individuals younger and older than 45 years of age was observed at all the three evaluated time points (Table S1). The difference in BMD values and >15% change measurements between males and females younger than 45 years was borderline significant (Table 4 and Table S1). Remarkably, despite the similarity in the administered steroid doses, vertebral BMD changes in male patients younger than 30 years were significantly less pronounced than in all other patients (Table 5).

The evaluation of median BMD changes between baseline and the end of treatment in correlation with the treatment protocol demonstrated significant differences for patients treated with ABVD × 2–4 versus ABVD × 6 (*p* = 0.000), for patients treated with EB × 2 + ABVD × 4 versus EB × 4–6 (*p* = 0.001) and for those receiving ABVD × 6 versus EB × 2 + ABVD × 4 (*p* = 0.001). However, the corresponding difference for patients treated with ABVD × 6 versus EB × 2–6 was non-significant (*p* = 0.108) (Table 2). A comparison of patients receiving EB × 2 + ABVD × 4 with those treated with EB × 4–6 demonstrated significantly greater mean BMD loss at the end of therapy in the former group. This could be attributed to a decade difference in the median age between the groups (39 versus 29, respectively; *p* = 0.06). The difference became non-significant at 6 months post-treatment because of a further BMD decrease in the latter group (Table 2).

At 6-months post-therapy, various patterns in the dynamics of bone mineral loss were observed. The aforementioned difference turned to non-significant in patients treated with ABVD × 2–4 compared to those treated with ABVD × 6 (*p* = 0.131). A comparison of patients receiving ABVD ×6 versus EB-containing regimens showed a highly significant difference in BMD loss between the two groups, equating to 11% versus 20%, respectively (*p* = 0.000) (Table 2).

### 3.3. The Incidence of Significant Vertebral BMD Decrease Assessed According to the Cutoff of 15%

A BMD reduction of >15% at the end of treatment compared to the baseline measurements was observed in 47.9% of all patients. The corresponding incidence among patients with advanced-stage and early-stage disease was 57 and 35.9%, respectively (*p* = 0.002) (Table 4).

In univariate analysis, age, the type of the treatment protocol, the number of treatment cycles, and a cumulative dose of HE per body surface >3400 mg/m^2^ were found to be associated with a significant vertebral BMD reduction at the end-of-treatment compared to baseline measurements (Table 4). The incidence of >15% BMD decrease at the end-of-treatment was significantly lower in male patients younger than 30 years than in all other patients (*p* = 0.018). These findings were persistent at 6 months post-therapy (*p* = 0.017). Furthermore, this incidence was significantly higher among males aged >45 years relative to males ≤45 years (*p* = 0.039).

The percentage of patients with a vertebral BMD decrease of >15% significantly differed between those treated with ABVD and EB (*p* = 0.038) (Table 3). This difference persisted at 6 months post-treatment, equating to 31% and 59%, respectively (*p* = 0.001). Patients who received dexamethasone and prednisone as part of EB ± ABVD protocols demonstrated a significantly higher incidence of >15% reduction in vertebral BMD compared to patients who received dexamethasone alone as part of their ABVD regimen both at the end of therapy and 6 months later (Table 4). Furthermore, at the end of therapy, this incidence was significantly increased in individuals treated with two cycles of EB + ABVD ×4 relative to patients who received 4–6 cycles of EB, but this difference became non-significant 6 months after treatment completion. Notably, the median age of the patients in the latter comparison was 39 years and 29 years, respectively, which could explain at least in part the observed differences (Table 4).

The results of the-end-of-therapy evaluation revealed osteopenia (≤120 HU) in a total of 14% of patients (*n* = 30): 10% (*n* = 15) of those treated with ABVD versus 23% (*n* = 15) of the ones receiving EB (*p* = 0.019) (Table 3). These findings remained persistent at 6 months post-therapy, equating to 14, 10, and 22%, respectively. However, due a decrease in the cohort size at 6 months, the difference between the cohorts did not reach statistical significance (*p* = 0.082). Remarkably, BMD loss of >25% both at the end of treatment and 6 months later compared to baseline was significantly more frequent among patients treated with EB (*p* = 0.000) (Table 3).

A sub-analysis of a potential correlation of various cumulative doses of HE per body surface and a >15% reduction in vertebral BMD identified 3400 mg/m^2^ as the dose associated with highly significant changes (*p* = 0.01) (Table 4 and Table S2).

A multivariate analysis for the prediction of vertebral BMD loss of >15%, based on the comparison of end-of-therapy and baseline data, identified the following parameters as significant risk factors: age ≥30 years for both genders and the female gender (HR = 2.27; 95% CI 1.17–4.41; *p* = 0.015) as well as chemotherapy protocols other than ABVD × 2–4 (ABVD × 6: HR = 7.03; 95% CI 2.95–16.76; *p* = 0.000; EB-containing regimens: HR = 7.91; 95% CI 3.15–19.85; *p* = 0.000). At 6 months after the end of therapy, patient demographics (HR = 2.75; 95% CI 1.14–6.65; *p* = 0.024) and the treatment with EB-containing regimens (HR = 5.02; 95% CI 1.86–13.52; *p* = 0.001) remained highly significant.

## 4. Discussion

The currently applied management of HL patients, based on both prognostic and predictive assessments, provides uniquely high PFS and OS rates as well as long life expectancy. These successful outcomes emphasize the importance of preventing long-term side effects of treatment, like chronic fatigue, secondary malignancies, cardiac toxicity, and BMD loss. The latter phenomenon is attributed not only to the detrimental impact of chemotherapy [22,28,29], but is actually multifaceted and related to patient age, the HL interference with bone homeostasis [30], therapy-induced hypogonadism in both males and females [31,32,33], and the use of glucocorticosteroids as part of either antiemetic or chemotherapy regimens [34,35].

Steroids are universally incorporated in antiemetic regimens, developed to help patients with chemotherapy-induced nausea and vomiting (CINV) [13,36,37]. According to the Cancer Care Alberta 2019 Endorsement of the 2017 ASCO Guidelines, highly emetogenic chemotherapy such as EB, may cause CINV in >90% of patients, while moderately emetogenic chemotherapy, like ABVD, leads to CINV in 30–90% of patients.

Yet, despite the clear beneficial effects of these agents, there is ample evidence of an association between steroid therapy and osteopenia/osteoporosis [16,38] in patients with various types of cancer [21,22,39,40,41]. The NCCN Task Force Reports emphasize the crucial need for the assessment and treatment of cancer therapy-related BMD loss, as an integral part of comprehensive cancer management [42,43].

The current study has demonstrated a >15% reduction in the vertebral BMD from the baseline to the end of therapy in as many as 48% of the evaluated patients, with a significant correlation between the steroid dosage and the degree of BMD loss. Specifically, the cumulative HE dose per body surface of >3400 mg/m^2^ has been consistently associated with a BMD decrease of >15% between these two time points. Moreover, these findings have remained persistent through the first 6 months following therapy completion in patients treated with EB-regimens, which raises significant concern and leaves physicians with a pressing need for further refinement in the risk-adapted management of HL patients. In fact, our data on BMD reduction after exposure to even moderate steroid doses call for reconsideration of the liberal use of these agents as antiemetics.

In this context, the correspondence between a particular degree of BMD decrease and a specific therapeutic protocol identified in the present study may have an important bearing on decision-making regarding the optimal HL treatment strategy. We have observed that among patients treated with ABVD only, the BMD reduction at the end of therapy has been comparable in those receiving 2–4 cycles of this regimen and patients treated with 6 ABVD cycles. Remarkably, 6 months later, substantial bone mineral recovery has occurred in patients from the latter group. This could be attributed to a lower cumulative dose of steroids added only as antiemetic therapy and a mild gonadotoxic effect of this regimen. In the case of patients treated with EB × 2 + ABVD × 4 versus those receiving EB × 4–6, unexpectedly, the mean BMD loss at the end of therapy has been found to be significantly higher in the former group. This finding could be related to a markedly older median age of those patients. Yet, this difference has turned to be non-significant at 6 months post-treatment due to a further BMD reduction in the latter group. At the same time, a comparison of patients receiving ABVD × 6 versus EB-containing regimens has shown a highly significant difference in the BMD loss between the two groups at 6 months of follow-up.

A large GHSG study has reported the 0.1% incidence of osteonecrosis in early-stage HL patients treated with 2–4 ABVD cycles relative to the 0.6–1% incidence in advanced-stage HL patients treated with 4–8 cycles of BEACOPP [44]. The median age of the patients included in that study has been 33 years, which is comparable to the age of individuals participating in our study (29 years). The GHSG analysis has also identified a high cumulative dose of corticosteroids, a young patient age, the male gender, and advanced-stage disease as risk factors for osteonecrosis. While patients receiving ABVD have not been given any steroids as per the treatment protocol, individuals exposed to EB regimens have received a median prednisone dose of 7300 mg (36,500 HE dose). The median HE dose given in our study to patients treated with EB-containing regimens has been 10,770, which is about one-third of the dose applied by the GHSG. Importantly, none of the patients treated with the EB protocol in the current study developed osteonecrosis, suggesting that our policy of reducing the steroid use duration to one week only, applied in the HL treatment, is beneficial. This conclusion is consistent with the suggestion by the GHSG to attempt decreasing corticosteroid doses given to HL patients [44].

The issue of treatment-induced BMD changes in patients with hematological malignancies has been addressed in a number of recent studies. Ruchlemer et al. have reported BMD loss in 181 hematological patients (median age 67.9 years), evaluated using dual-energy X-ray absorptiometry (DXA) [28]. A T-score reduction has been found in 65% of those patients, 38% of whom had osteopenia, and 27% had osteoporosis.

Along the same lines, a small prospective study from France, using DXA for BMD assessment, has reported significant BMD reduction and osteoporotic fractures post-chemotherapy in 41 lymphoma patients (median age 59 years) [29]. Furthermore, a multicenter prospective study evaluating with DXA the bone remodeling prior to and after chemotherapy in 61 newly-diagnosed NHL patients [45] has shown a more pronounced post-chemotherapy BMD decline in males and individuals above 55 years of age.

A study applying CT for the estimation of the R-CHOP (rituximab, cyclophosphamide, doxorubicin, vincristine, prednisone) protocol impact on the BMD in 111 DLBCL patients [21] has demonstrated an average 14% BMD reduction post-therapy, which has remained persistent through the following 2 years. Furthermore, in 14% of the patients, vertebral compression fractures have been identified by CT during the follow-up, emphasizing the detrimental role of glucocorticoid-induced osteoporosis in patient survivorship.

A large, recent Danish Nationwide cohort study, including 2589 patients with DLBCL or follicular lymphoma, has reported 5- and 10-year cumulative risks of osteoporotic events in lymphoma patients of 10.0 and 16.3%, respectively, relative to only 6.8 and 13.5% in a matched general population cohort [46]. The investigators have concluded that high-dose steroids included in standard anti-lymphoma protocols elevate the osteoporotic risk during the first 2 years post-treatment. The findings of the present study are consistent with these data and provide further evidence advocating for the feasibility of using PET/CT results for the evaluation of BMD dynamics and the effect of treatment protocols on the bone mineral status of HL patients.

The EB protocol, comprising two gonadotoxic alkylating agents, cyclophosphamide and procarbazine, is applied in the management of HL and is associated with higher PFS than ABVD. However, this often comes at the expense of increased treatment-related gonadotoxicity [47,48] that is an established contributing factor to reduced BMD. As early as 4 decades ago, Waxman et al. revealed long-term gonadotoxicity of the MOPP (nitrogen mustard, vinblastine, prednisolone, procarbazine) regimen in patients with advanced-stage HL [31]. At a mean follow-up of 6.9 years, a reduced gonadal function was observed in 78% of 46 males and 28 females. Holmes et al., evaluating BMD at 3.4 years post-therapy, in 29 young HL male patients treated with a procarbazine-containing regimen, pointed to a potential association between hypogonadism secondary to the use of gonadotoxic agents, high doses of steroids, HL per se, and BMD loss in this patient setting [32]. Likewise, a study from the Norwegian Radium Hospital, with a median follow-up of 15 years, assessing the gonadal function in a total of 294 male lymphoma patients, including 165 HL survivors (a median age at diagnosis—33 years), reported a significantly increased risk for exocrine hypogonadism in all the treatment groups, apart from that receiving ABVD [33]. Moreover, at the time of the survey, patient age above 50 years was found to be associated with a 5-time higher risk of endocrine hypogonadism than the age <40 years.

Overall, the results of the above-referenced studies focusing on gonadotoxicity of HL-treatment protocols and the effect of steroids on BMD are compatible with the findings of our study, demonstrating that regimens containing alkylating agents and steroids are prone to cause hypogonadism and osteopenia even in a relatively young age. Of note, in the UK, due to the gonadotoxicity of EB, it has been substituted with procarbazine-free escalated BEACOPDAC, containing dacarbazine [49]. A similar approach is being currently investigated in the ongoing GHSG HD21 trial, comparing EB with BrECADD (brentuximab vedotin, etoposide, cyclophosphamide, doxorubicin, dacarbazine, and dexamethasone [50,51].

Similar to the present study, a deleterious effect of patient age and the steroid dosage used during treatment on the BMD loss has been recently reported by Cohen et al. in a cohort of 80 HL patients treated with ABVD or EB-containing regimens [22]. That group has also employed PET/CT results for BMD assessment and observed a mean of 14% ± 10 decline in this parameter at the end of therapy, with a mean 4.6% ± 10.4 improvement during the 14-month follow-up. In multivariate analysis, low baseline BMD has been identified as the only parameter significantly associated with a further BMD decrease.

To the best of our knowledge, the current study is the first to suggest a multivariate model evaluating vertebral BMD loss in correlation to HL therapeutic regimens. Using this model, age ≥30 years for both genders, the female gender, and chemotherapy protocols other than ABVD × 2–4 have been identified as significant risk factors for a BMD reduction of >15% at the end of therapy relative to the baseline level. At 6 months after the therapy completion, only patient demographics and the use of EB-containing protocols persisted in being highly significant. The differences in the BMD levels between the treatment groups observed at the follow-up assessment could be related to the cumulative dose of steroids employed and gonadotoxicity of the regimens.

This study has several limitations, both related and unrelated to its retrospective nature. Non-contrast enhanced CT scans have been used as a surrogate for DXA in BMD evaluations. However, this opportunistic approach has been previously validated and published [23,27]. Patients in the EB-treated group have been significantly older than those included in the ABVD ×6-treated group [median age 33 (18–57) versus 28 (18–59); *p* = 0.003], that could contribute to a higher rate of BMD loss in the former cohort. However, there have been no significant intergroup differences in the BMD levels, either at baseline or at the end of treatment, making this deficiency minor. Furthermore, since EB regimens include both alkylating agents and steroids, we could not separately evaluate the effects of each of these components on patient BMD. Additionally, advanced-stage patients with IPS ≥3 have received EB-containing treatments, while those with IPS 0–2 had initiated therapy with ABVD and 18% of them have been switched to EB based on PET-2 results. This precluded the assessment of the impact of HL per se on BMD, in correlation with IPS, within the subgroup of patients with advanced disease.

## 5. Conclusions

The current study demonstrates a significant treatment-related reduction in the vertebral BMD of HL patients above the age of 30 years, which may lead to osteopenia and osteoporosis. The use of EB-including protocols is found to be associated with a significantly greater rate of BMD loss than ABVD, due to the gonadotoxic effect of the former regimen and the high dose of steroids incorporated in it. A restrictive approach to the use of glucocorticoids and substitution of gonadotoxic agents, like procarbazine, with non-gonadotoxic drugs, like dacarbazine, should be considered in this patient population. Continuous assessment of the BMD status and early intervention, such as maintaining adequate vitamin D levels to preserve their bone tissue, is warranted in HL survivors, particularly those receiving EB regimens.

## Figures and Tables

**Table 1:** Patient characteristics according to treatment protocols and BMD measurements.

Evaluated Parameters		Total	ABVD	EB ± ABVD	Chi-Square *p*-Value
		No. of pts (%)	No. of pts (%)	No. of pts (%)	
**All patients**		213 (100)	147 (100)	66 (100)	
**Gender**	Male	105 (49)	63 (43)	42 (64)	0.286
	Female	108 (51)	84 (57)	24 (36)	
**Age (years)**	<30	113 (53)	82 (56)	31 (47)	0.058
	30–45	68 (32)	50 (34)	18 (27)	
	>45	32 (15)	15 (10)	17 (26)	
**HL stage**	I	12 (6)	10 (7)	2 (3)	0.000
	II	120 (57)	100 (69)	20 (30)	
	III	38 (18)	28 (19)	10 (15)	
	IV	41 (19)	7 (5)	34 (52)	
**HL risk group**	Early-stage, favorable	11 (5)	11 (7)		0.000
	Early-stage, unfavorable	81 (38)	79 (54)	2 (3)	
	Advanced-stage, standard risk (IPS 0–2)	66 (31)	54 (37)	12 (18)	
	Advanced-stage, high risk (IPS 3–7)	55 (26)	3 (2)	52 (79)	
**Glucocorticoids used**	Dexamethasone	148 (69)	147 (100)	1 (2)	0.84
	Dexamethasone + prednisone	65 (31)		65 (98)	
**Cumulative HE dose per body surface (mg/m^2^)**	≤2100	24 (11)	24 (16)		0.000
	2100.1–3400	73 (34)	72 (49)	1 (2)	
	≥3400	116 (54)	51 (35)	65 (98)	
Baseline BMD *	Normal ≥ 160 HU	173 (81)	118 (80)	55 (83)	0.614
	Borderline, 121–159 HU	31 (15)	21 (14)	10 (15)	
	Osteopenia, 80–120 HU	8 (4)	7 (5)	1 (2)	
	Osteoporosis, <80 HU	1 (0)	1 (1)		

* HL staging PET/CT (non-IV contrast CT) scans were used to measure the baseline vertebral BMD at the L3 level. BMD: bone mineral density; no. of pts: number of patients; HL: Hodgkin lymphoma; IPS: International Prognostic Score; HE: hydrocortisone equivalent; HU: Hounsfield units; ABVD: adriamycin, bleomycin, vinblastine, dacarbazine; EB: escalated BEACOPP, including bleomycin, etoposide, adriamycin, cyclophosphamide, oncovin, procarbazine, prednisone.

**Table 2:** The evaluation of BMD changes between baseline and the end of treatment in correlation with the treatment protocol and the cumulative steroid dose used.

Evaluated Parameters	TOTAL		ABVD × 2–4 vs. ABVD × 6		EB × 2 + ABVD × 4 vs. EB × 4–6		ABVD × 6 vs. EB × 2 + ABVD × 4		ABVD × 6 vs. EB 2–6	
	No. of pts	Median (Range)	No. of pts: 48 vs. 99	*p*-Value	No. of pts: 38 vs. 28	*p*-Value	No. of pts: 99 vs. 38	*p*- Value	No. of pts: 99 vs. 66	*p*-Value
**Age, years**, median (range)	213	29 (18–59)	30 (19–58) vs. 28 (18–59)	0.079	39 (19–57) vs. 29 (18–56)	0.065	28 (18–59) vs. 39 (19–57)	0.001	28 (18–59) vs. 33 (18–57)	0.003
Baseline BMD *, HU, median (range)	213	197 (30.5–320)	187 (30.5–238.5) vs. 210 (92–290.8)	0.001	192 (118.2–320) vs. 215 (122.7–316.3)	0.08	210 (92–290.8) vs. 192 (118.2–320)	0.097	210 (92–290.8) vs. 197 (118.2–320)	0.322
EOT BMD **, HU, median (range)	213	165 (28–277.3)	173 (27.5–257.3) vs. 165 (80.4–26)	0.828	139 (64.3–277.3) vs. 183 (74.5–270)	0.003	165 (80.4–26) vs. 139 (64.3–277.3)	0.001	165 (80.4–26) vs. 160 (64.3–277.3)	0.159
**ΔbetweenEOT and baseline BMD, %,** median (range)	213	15 (0–54)	9 (0–23) vs. 19 (0–45.9)	0.000	27 (0–52) vs. 13 (0–54.6)	0.001	19 (0–45.9) vs. 27(0–52)	0.001	19 (0–45.9) vs. 18 (0–54.6)	0.108
6 months post-EOT BMD ***, HU, median (range)			**No. of pts: 35 vs. 75**		**No. of pts: 31 vs. 20**		**No. of pts: 75 vs.31**		**No. of pts: 75 vs. 51**	
	161	169 (23.9–258.3)	162 (24–258.3) vs. 183 (81.3–264.7)	0.074	139 (69.3–275.7) vs. 164 (87.3–301)	0.293	183 (81.3–264.7) vs. 139 (69.3–275.7)	0.007	183 (81.3–264.7) vs. 154 (69.3–301)	0.007
**Δbetween6 months post-EOT andbaseline BMD, %,** median (range)	161	12 (0–49.8)	7 (0–23.7) vs. 11 (0–41.3)	0.131	16.0 (0–49.8) vs. 23.0 (0–39.5)	0.728	11(0–41.3) vs. 16 (0–49.8)	0.002	11(0–41.3) vs. 20 (0–49.8)	0.000
**Cumulative HE dose, mg,** median (range)			**No. of pts: 48 vs. 99**		**No. of pts: 38 vs. 28**		**No. of pts: 99 v 38**		**No. of pts: 99 vs. 66**	
	213	6000 (1600–22,400)	4000 (1600–4000) vs. 6000 (3000–6000)	0.000	10,200 (7260–1760) vs. 16,140 (6000–22,400)	0.000	6000 (3000–6000) vs. 10,200 (7260–1760)	0.000	6000 (3000–6000) vs. 10,770 (6000–22,400)	0.000
	213	3468 (952–11,546)	2116 (952–2597) vs. 3409 (1744–4839)	0.000	5668 (4132–10,353) vs. 8691 (2970–11,546)	0.000	3409 (1744–4839) vs. 5668 (4132–10,353)	0.000	5668 (4132–10,353) vs. 6025 (2970–11,546)	0.000
**Cumulative HE dose per surface area, mg/m^2^,** median (range)	213	3468 (952–11,546)	2116 (952–2597) vs. 3409 (1744–4839)	0.000	5668 (4132–10,353) vs. 8691 (2970–11,546)	0.000	3409 (1744–4839) vs. 5668 (4132–10,353)	0.000	5668 (4132–10,353) vs. 6025 (2970–11,546)	0.000

* HL staging PET/CT (non-IV contrast CT) scans were used to measure the baseline vertebral BMD at the L3 level. ** HL PET/CT (non-IV contrast CT) scans performed at the end of treatment were used to measure vertebral BMD at that time point. *** HL PET/CT (non-IV contrast CT) scans performed 6 months after the end of treatment were used to measure vertebral BMD at that time point. BMD: bone mineral density; pts: patients; Δ: difference; HE: hydrocortisone equivalent; HU: Hounsfield units; ABVD: adriamycin, bleomycin, vinblastine, dacarbazine; EB: escalated BEACOPP, including bleomycin, etoposide, adriamycin, cyclophosphamide, oncovin, procarbazine, prednisone.

**Table 3:** Patient BMD evaluated at the end of treatment and 6 months later.

Evaluated Parameters		Total	ABVD	EB ± ABVD	*p*-Value
		No. of pts (%)	No. of pts (%)	No. of pts (%)	
EOT * BMD (HU)	Normal ≥ 160	125 (59)	92 (63)	33 (50)	0.054
	Borderline, 121–159	58 (27)	40 (27)	18 (27)	
	Osteopenia, 80–120	26 (12)	14 (10)	12 (18)	
	Osteoporosis, <80	4 (2)	1 (1)	3 (5)	
BMD at 6 months post-EOT ** (HU)	Normal ≥ 160	101(63)	77 (70)	24 (47)	0.034
	Borderline, 121–159	38 (24)	22 (20)	16 (31)	
	Osteopenia, 80–120	19 (12)	10 (9)	9 (18)	
	Osteoporosis, <80	3 (2)	1 (1)	2 (4)	
EOT * osteopenia **(≤120 HU)**	No	183 (86)	132 (90)	51 (77)	0.019
	Yes	30 (14)	15 (10)	15 (23)	
Osteopenia at 6 months post-EOT ** (≤120 HU)	No	139 (86)	99 (90)	40 (78)	0.082
	Yes	22 (14)	11 (10)	11 (22)	
**Δ** **between** **EOT and baseline BMD (%)**	≤15	111 (52)	84 (57)	27 (41)	0.038
	>15	102 (48)	63 (43)	39 (59)	
**Δ** **between** **EOT and baseline BMD (%)**	≤25	170 (80)	128 (87)	42 (64)	0.000
	>25	43 (20)	19 (13)	24 (36)	
**Δ** **between** **6 months post-EOTand** **baseline BMD (%)**	≤15	97 (60)	76 (69)	21 (41)	0.001
	>15	64 (40)	34 (31)	30 (59)	
**Δ** **between** **6 months post-EOTand** **baseline BMD (%)**	≤25	139 (86)	107 (97)	32 (63)	0.000
	>25	22 (14)	3 (3)	19 (37)	

* HL PET/CT (non-IV contrast CT) scans performed at the end of treatment were used to measure vertebral BMD at that time point. ** HL PET/CT (non-IV contrast CT) scans performed 6 months after the end of treatment were used to measure vertebral BMD at that time point. BMD: bone mineral density; no. of pts: number of patients; EOT: end-of-treatment; HU: Hounsfield units; Δ: difference; ABVD: adriamycin, bleomycin, vinblastine, dacarbazine; EB: escalated BEACOPP, including bleomycin, etoposide, adriamycin, cyclophosphamide, oncovin, procarbazine, prednisone.

**Table 4:** Factors that influence BMD decrease of >15% between baseline and end of treatment, or between baseline and 6 months post-treatment.

Evaluated Parameters		Total	BMD Decrease >15% between Baseline and End of Treatment			Total	BMD Decrease >15% between Baseline and 6 Months Post-Treatment		
		No. of pts	No. of pts (%)	*p*-Value	OR	No. of pts	No. of pts (%)	*p*-Value	OR
**All patients**		213	102 (47.9)			161	64 (39.8)		
**Gender**	Males	105	46 (43.8)		1.0	74	28 (37.8)		1.0
	Females	108	56 (51.9)	0.241	1.38 (0.8–2.4)	87	36 (41.4)	0.647	1.16 (0.6–2.2)
**Age group, years**	<30	113	48 (42.5)		1.0	79	24 (30.4)		1.0
	30–45	68	36 (52.9)	0.173	1.52 (0.8–2.8)	57	23 (40.4)	0.229	1.55 (0.8–3.2)
	>45	32	18 (56.3)	0.170	1.74 (0.8–3.8)	25	17 (68.0)	0.001	4.87 (1.9–12.8)
**Age and gender distribution, with data on males ≤45 y.o. used as reference**	Males ≤ 45	83	32 (38.6)		1.0	56	16 (28.6)		1.0
	Females ≤ 45	98	52 (53.1)	0.052	1.8 (1–3.3)	80	31 (38.8)	0.221	1.58 (0.8–3.3)
	Males > 45	22	14 (63.6)	0.039	2.79 (1.1–7.4)	18	12 (66.7)	0.006	5 (1.6–15.6)
	Females > 45	10	4 (40.0)	0.929	1.06 (0.3–4.1)	7	5 (71.4)	0.039	6.25 (1.1–35.6)
**Age and gender distribution, with data on males <30 y.o. used as reference**	Males < 30	58	20 (34.5)		1.0	36	8 (22.2)		1.0
	Females < 30	55	28 (50.9)	0.079	1.97 (0.9–4.2)	43	16 (37.2)	0.153	2.07 (0.8–5.6)
	Males ≥ 30	47	26 (55.3)	0.034	2.35 (1.1–5.2)	38	20 (52.6)	0.008	3.89 (1.4–10.7)
	Females ≥ 30	53	28 (52.8)	0.053	2.13 (1–4.6)	44	20 (45.5)	0.033	2.92 (1.1–7.8)
	Males < 30	58	20 (34.5)		1.0	36	8 (22.2)		1.0
	All others	155	82 (52.9)	0.018	2.13 (1.1–4)	125	56 (44.8)	0.017	2.84 (1.2–6.7)
**HL stage**	Early	92	33 (35.9)		1.0	70	18 (25.7)		1.0
	Advanced	121	69 (57.0)	0.002	2.37 (1.4–4.1)	91	46 (50.5)	0.002	2.95 (1.5–5.8)
**Comparison of all treatment protocols**	ABVD × 2–4	48	8 (16.7)		1.0	35	9 (25.7)		1.0
	ABVD × 6	99	55 (55.6)	0.000	6.25 (2.7–14.7)	75	25 (33.3)	0.422	1.44 (0.6–3.5)
	EB × 2–6	66	39 (59.1)	0.000	7.22 (2.9–17.8)	51	30 (58.8)	0.003	4.13 (1.6–10.6)
**Comparison of EB-containing protocols**	EB × 2 + ABVD × 4	38	29 (76.3)		1.0	31	17 (54.8)		1.0
	EB × 4–6	28	10 (35.7)	0.001	0.17 (0.1–0.5)	20	13 (65.0)	0.473	1.53 (0.5–4.9)
**Glucocorticoids used**	Dexamethasone	148	63 (42.6)		1.0	111	34 (30.6)		1.0
	Dexamethasone and prednisone	65	39 (60.0)	0.020	2.02 (1.1–3.7)	50	30 (60.0)	0.001	3.4 (1.7–6.8)
**Cumulative HE dose** **per body surface (mg/m^2^)**	≤3400	97	31 (32.0)		1.0	73	21 (28.8)		1.0
	>3400	116	71 (61.2)	0.000	3.36 (1.9–5.9)	88	43 (48.9)	0.01	2.37 (1.2–4.6)

BMD: bone mineral density; no. of pts: number of patients; OR: odds ratio; HL: Hodgkin lymphoma; EOT: end-of-treatment; HU: Hounsfield units; HE: hydrocortisone equivalent; ABVD: adriamycin, bleomycin, vinblastine, dacarbazine; EB: escalated BEACOPP, including bleomycin, etoposide, adriamycin, cyclophosphamide, oncovin, procarbazine, prednisone.

**Table 5:** BMD changes in male patients younger than 30 years compared to all other patients.

Evaluated Parameters	Gender/Age	No. of pts	Mean (±SD)	Median (Range)	Mann–Whitney *p*-Value
**Age (years)**	Males < 30 y.o.	58	24.4 (3.3)	24 (18–29)	
	All others	155	35.2 (11.5)	33 (18–59)	0.000
Baseline BMD * (HU)	Males < 30 y.o.	58	207.0 (31.5)	203.0 (110–273.8)	
	All others	155	193.9 (46.2)	193.5 (30.5–320)	0.047
EOT BMD ** (HU)	Males < 30 y.o.	58	180.8 (28.9)	180.2 (115.4–267)	
	All others	155	159.8 (42.6)	161.0 (27.5–277.3)	0.000
6 months post-EOT BMD *** (HU)	Males < 30 y.o.	36	186.6 (38.7)	192.4 (111.8–275.7)	
	All others	125	164.7 (46)	165.7 (23.9–301)	0.015
**Δ** **between** **EOT and baseline BMD (%)**	Males < 30 y.o.	58	13.1 (9.2)	12.6 (0–31.5)	
	All others	155	17.6 (11.6)	17.2 (0–54.6)	0.015
**Δ** **between** **6 months post-EOT and** **baseline BMD (%)**	Males < 30 y.o.	36	8.8 (10.6)	5.6 (0–39.5)	
	All others	125	15.3 (11.7)	14.2 (0–49.8)	0.002
**Cumulative HE dose (mg)**	Males < 30 y.o.	58	8500 (5220)	6000 (3000–21800)	
	All others	155	7220 (3840)	6000 (1600–22400)	0.292
**Cumulative HE dose per body surface (mg/m^2^)**	Males < 30 y.o.	58	4470 (2600)	3343 (1740–10620)	
	All others	155	4110 (2180)	3500 (950–11540)	0.864

* HL staging PET/CT (non-IV contrast CT) scans were used to measure the baseline vertebral BMD at the L3 level. ** HL PET/CT (non-IV contrast CT) scans performed at the end of treatment were used to measure vertebral BMD at that time point. *** HL PET/CT (non-IV contrast CT) scans performed 6 months after the end of treatment were used to measure vertebral BMD at that time point. BMD: bone mineral density; pts: patients; EOT: end-of-treatment; HU: Hounsfield units; Δ: difference; HE: hydrocortisone equivalent; y.o.: years old.

## Data Availability

The data that support the findings of this study are available from the corresponding author upon reasonable request.

## Supplementary Materials

The following are available online at https://www.mdpi.com/article/10.3390/cancers14030495/s1, Table S1: Vertebral BMD at baseline, end of therapy and 6-months post-therapy, analyzed according to patient gender and age cutoff of 45 years. Table S2: BMD decrease of >15% between baseline and the end of treatment according to a cumulative hydrocortisone equivalent dose per body surface used in different treatment protocols: Bi-variate log regression analysis.
