# Supplementary material for: Reduction of the Vertebral Bone Mineral Density in Patients with Hodgkin Lymphoma Correlates with Their Age and the Treatment Regimen They Received
Source: Cancers (Basel). 2022 Jan 19;14(3):495. doi: 10.3390/cancers14030495 (PMC8833719; doi:10.3390/cancers14030495)
Supplement: Supplementary file 1 [file cancers-14-00495-s001.zip › cancers-1546300-supplementary.pdf]

Supplementary Materials

Table S1. Vertebral BMD at baseline, end of therapy and 6-months post-therapy analyzed according to patient gender and age cutoff of 45 years.

Evaluated Parameters	Median (Range)	Females <45 Years vs. Males <45 Years		Males & Females ≥45 Years vs. Males <45 Years		Males & Females ≥45 Years vs. Females <45 Years	
	**No. of pts 213**	**No. of pts 98 vs. 83**	***p*-Value**	**No. of pts 32 vs. 83**	***p*-Value**	**No. of pts: 32 vs. 98**	***p*-Value**
**Age, years, median (range)**	29 (18–59)	28 (18–45) vs. 27 (18–45)	0.488	54 (47–59) vs. 27 (18–45)	0.000	54 (47–59) vs. 28 (18–45)	0.000
**Baseline BMD *, HU, median (range)**	196.8 (30.5–320)	212.9 (30.5–320) vs. 200.4 (101.2–273.8)	0.073	138.9 (92–192.9) vs. 200.4 (101.2–273.8)	0.000	138.9 (92–192.9) vs. 212.9 (30.5–320)	0.000
**EOT BMD **, HU, median (range)**	164.9 (27.5–277.3)	170.6 (27.5–277.3) vs. 174.1 (74.5–267)	0.807	112.5 (64.3–172.5) vs. 174.1 (74.5–267)	0.000	112.5 (64.3–172.5) vs. 170.6 (27.5–277.3)	0.000
**Δ between EOT and baseline BMD, %, median (range)**	15.4 (0–54.6)	17.9 (0–42.1) vs. 13 (0–54.6)	0.107	17.1 (0–52.1) vs. 13 (0–54.6)	0.015	17.1 (0–52.1) vs. 17.9 (0–42.1)	0.192
	**No. of pts 161**	**No. of pts 80 vs. 56**	***p*-value**	**No. of pts 25 vs. 56**	***p*-value**	**No. of pts 25 vs. 80**	***p*-value**
**6 months post-EOT BMD *** HU, median (range)**	169 (23.9–301)	181 (23.9–301) vs. 176.8 (102–275.7)	0.377	110 (69.3–168.7) vs. 176.8 (102–275.7)	0.000	110 (69.3–168.7) vs. 181 (23.9–301)	0.000
**Δ between 6 months post-EOT and baseline BMD, %, median (range)**	11.7 (0–49.8)	12.9 (0–37.9) vs. 8.6 (0–41.3)	0.194	23.7 (0–49.8) vs. 8.6 (0–41.3)	0.000	23.7 (0–49.8) vs. 12.9 (0–37.9)	0.000
	**No. of pts 213**	**No. of pts 98 vs. 83**	***p*-value**	**No. of pts 32 vs. 83**	***p*-value**	**No. of pts: 32 vs. 98**	***p*-value**
**Cumulative HE dose, mg, median (range)**	6000 (1600–22,400)	6000 (2000–19,140) vs. 6000 (1600–22,400)	0.145	7325 (2000–20,040) vs. 6000 (1600–22,400)	0.802	7325 (2000–20,040) vs. 6000 (2000–19,140)	0.101
**Cumulative HE dose per body surface, mg/m², median (range)**	3468 (952–11,546)	3529 (1,170–10,950) vs. 3243 (952–11,546)	0.365	4152 (995–10,277) vs. 3243 (952–11,546)	0.958	4152 (995–10,277) vs. 3529 (1,170–10,950)	0.785

* HL staging PET/CT (non-IV contrast CT) scans were used to measure the baseline vertebral BMD at the L3 level. ** HL PET/CT (non-IV contrast CT) scans performed at the end of treatment were used to measure vertebral BMD at that time point. *** HL PET/CT (non-IV contrast CT) scans performed 6 months after the end of treatment were used to measure vertebral BMD at that time point. Mann-Whitney *p*-value; BMD: bone mineral density; no. of pts: number of patients; EOT: end-of-treatment; HU: Hounsfield units; Δ: difference; HE: hydrocortisone equivalent; y.o.: years old.

Table S2. BMD decrease of >15% between baseline and the end of treatment according to a cumulative hydrocortisone equivalent dose per body surface used in different treatment protocols: Bi-variate log regression analysis.

	HE Dose per Surface Area (mg/m^2)	No. of Patients	Patients with BMD Decrease of >15%		
			no. (%)	*p*-value	OR
**Cumulative HE dose per surface area (mg/m^2)**	≤2100	24	5 (20.83)	0.000	1.00
	2100.1–3400	73	26 (35.62)	0.184	2.1 (0.7–6.3)
	>3400	116	71 (61.21)	0.001	6 (2.1–17.2)
**Males ≤45 y.o.**	≤2100	12	1 (8.33)	0.091	1.00
	2100.1–3400	34	13 (38.24)	0.082	6.81 (0.8–59.1)
	>3400	37	18 (48.65)	0.032	10.42 (1.2–89.1)
**Males >45 y.o. and all females**	≤2100	12	4 (33.33)	0.001	1.00
	2100.1–3400	39	13 (33.33)	1	1 (0.3–3.9)
	>3400	79	53 (67.09)	0.033	4.08 (1.1–14.8)
**ABVD ×2–4**	≤1800	8	1 (12.50)	0.472	1.00
	1800.1–2100	15	4 (26.67)	0.443	2.55 (0.2–27.7)
	<2100	25	3 (12.00)	0.97	0.96 (0.1–10.7)
**ABVD ×6**	≤2600	3	1 (33.33)	0.298	1.00
	2600.1–3399	45	22 (48.89)	0.607	1.91 (0.2–22.6)
	<3400	51	32 (62.75)	0.335	3.37 (0.3–39.7)
**EB ×2 + ABVD ×4**	≤5600	17	13 (76.47)		1.00
	>5600	21	16 (76.19)	0.984	0.99 (0.2–4.4)
**EB ×4–6**	≤8000	9	4 (44.44)		1.00
	>8000	19	6 (31.58)	0.509	0.58 (0.1–3)
**EB ×2–6**	≤5600	18	13 (72.22)	0.012	1.00
	5600–8000	28	19 (67.86)	0.754	0.81 (0.2–3)
	>8000	20	7 (35.00)	0.025	0.21 (0.1–0.8)

BMD: bone mineral density; HE: hydrocortisone equivalent; y.o.: years old; OR: odds ratio; ABVD: adriamycin, bleomycin, vinblastine, dacarbazine; EB: escalated BEACOPP, including bleomycin, etoposide, adriamycin, cyclophosphamide, oncovin, procarbazine, prednisone.
